# Mix-and-extrude: high-viscosity sample injection towards time-resolved protein crystallography

**DOI:** 10.1107/S1600576723004405

**Published:** 2023-06-12

**Authors:** Mohammad Vakili, Huijong Han, Christina Schmidt, Agnieszka Wrona, Marco Kloos, Iñaki de Diego, Katerina Dörner, Tian Geng, Chan Kim, Faisal H. M. Koua, Diogo V. M. Melo, Mathieu Rappas, Adam Round, Ekaterina Round, Marcin Sikorski, Joana Valerio, Tiankun Zhou, Kristina Lorenzen, Joachim Schulz

**Affiliations:** a European XFEL GmbH, Holzkoppel 4, Schenefeld 22869, Germany; b Sosei Heptares, Steinmetz Building, Granta Park, Great Abington, Cambridge CB21 6DG, United Kingdom; c Diamond Light Source, Harwell Science and Innovation Campus, Didcot OX11 0DE, United Kingdom; d Research Complex at Harwell, Rutherford Appleton Laboratory, Didcot OX11 0QX, United Kingdom; Uppsala University, Sweden; The European Extreme Light Infrastucture, Czech Republic

**Keywords:** time-resolved serial crystallography, mix-and-extrude, 3D-printed nozzles, membrane proteins, X-ray free-electron lasers

## Abstract

Time-resolved crystallography enables the visualization of protein molecular motion during a reaction. Although light is often used to initiate reactions in time-resolved crystallography, only a small number of proteins can be activated by light. However, many biological reactions can be triggered by the interaction between proteins and ligands. The sample delivery method presented here uses a mix-and-extrude approach based on 3D-printed microchannels in conjunction with a micronozzle. The diffusive mixing enables the study of the dynamics of samples in viscous media. The device design allows mixing of the ligands and protein crystals in 2 to 20 s. The device characterization using a model system (fluorescence quenching of iq-mEmerald proteins by copper ions) demonstrated that ligand and protein crystals, each within lipidic cubic phase, can be mixed efficiently. The potential of this approach for time-resolved membrane protein crystallography to support the development of new drugs is discussed.

## Abstract 2

3D-printed mixing high-viscosity extruder devices address time-resolved membrane protein crystallography challenges via compact dual-flow lipidic cubic phase injection.

## 1. Introduction

Membrane proteins comprise about 23% of all proteins (Uhlén *et al.*, 2015 ▸). They play an essential role in biological function and are the target of numerous medications on the market (Overington *et al.*, 2006 ▸). Owing to sample preparation challenges, the number of membrane protein structures that have been determined is rather low despite their biological significance. For rational drug design, the structures of the target protein in the apo and ligand-bound forms are essential to understand the mode of action.

By employing lipidic cubic phase (LCP) as a medium, membrane protein crystallization was facilitated (Landau & Rosenbusch, 1996 ▸; Li & Caffrey, 2020 ▸). LCP features a unique lipid bilayer and aqueous channel structure that is continuous, folded and highly curved. During crystallization, the cubic phase is locally converted to the lamellar phase by equilibrating with precipitant solutions, and the protein is concentrated in the lamellar phase to form a nucleus and ultimately a crystal (Cherezov & Caffrey, 2007 ▸; Caffrey, 2008 ▸). Multiple characterization studies have demonstrated the mobility of chemicals and proteins in LCP via lipidic or aqueous phases (Cherezov *et al.*, 2006 ▸; Li *et al.*, 2017 ▸; Li & Caffrey, 2011 ▸; Clogston & Caffrey, 2005 ▸; Eriksson & Lindblom, 1993 ▸; Boland *et al.*, 2018 ▸), indicating that LCP can be used as a medium for biochemical/biophysical characterization in addition to crystallization of membrane proteins.

Sample delivery for time-resolved serial crystallography with light-sensitive protein crystals is not different from those for serial crystallography for static structure determination; the only difference in the setup is the additional light source, often a laser, to initiate the reaction. Time-resolved studies of ligand binding, on the other hand, need an additional liquid channel for the ligand. For crystals grown in aqueous solution, mix-and-inject schemes using liquid jets (Pandey *et al.*, 2021 ▸; Hejazian *et al.*, 2020 ▸; Calvey *et al.*, 2016 ▸) or adding/injecting ligands on top of crystals for fixed target, drop-on-demand and tape-drive approaches have been developed as sample delivery methods for time-resolved serial crystallography (Mehrabi *et al.*, 2019 ▸; Butryn *et al.*, 2021 ▸; Beyerlein *et al.*, 2017 ▸).

The high-viscosity extruder (HVE) created by Weierstall *et al.* (2014 ▸) is one of the most widely used sample delivery methods for membrane protein crystals in serial crystallography. Within this HVE, a hydraulic plunger is used to amplify the pressure provided by an HPLC pump 14 times. This can provide pressure up to 10 000 psi to drive the sample from the sample reservoir into the capillary and the nozzle. Compared with glass syringes, which can withstand pressures of up to 1000 psi, this type of injection is more reliable for delivering viscous samples (Grünbein & Kovacs, 2019 ▸).

3D printing using two-photon polymerization (2PP) enables a rapid, reproducible and high-throughput nozzle fabrication, in addition to design flexibility (Knoška *et al.*, 2020 ▸; Nelson *et al.*, 2016 ▸). Recently, we presented our portfolio of 3D-printed sample delivery devices, including viscous extrusion tips that provide controllable sample streams (Vakili *et al.*, 2022 ▸) and are fully compatible with the well established Weierstall and co-workers HVE injection hardware. Here, we introduce the 3D-printed mix-and-extrude device, designed for the simultaneous use of two HVE setups. The device provides a second capillary port for introducing a ligand dispersed in viscous medium. With this, the mixing of two samples immediately before X-ray probing can be achieved. Thus, the mixing-HVE has great potential in enabling the time-resolved crystallographic study of membrane proteins for the investigation of ligand-binding processes.

Aside from their usage at an FEL reported here, our devices are also suited to the millisecond exposures used at synchrotrons. As our 3D-printed nozzles (both with and without mixing capabilities) are fully compatible with Weierstall’s HVE injector – they use the same standard 1/16′′ outer diamter (OD) steel tubings as the connective part – and our flow velocity regime (0.25–5.0 mm s^−1^) for stable viscous extrusion matches the parameters from previous serial synchrotron crystallography experiments (Botha *et al.*, 2015 ▸, 2018 ▸; Nogly *et al.*, 2015 ▸; Weinert *et al.*, 2017 ▸), our nozzles represent promising alternatives to the conventionally utilized ground glass capillary tips that are prone to manual error and lack device reproducibility.

## 2. Materials and methods

### 2.1. Design choices

**Figure 1:**
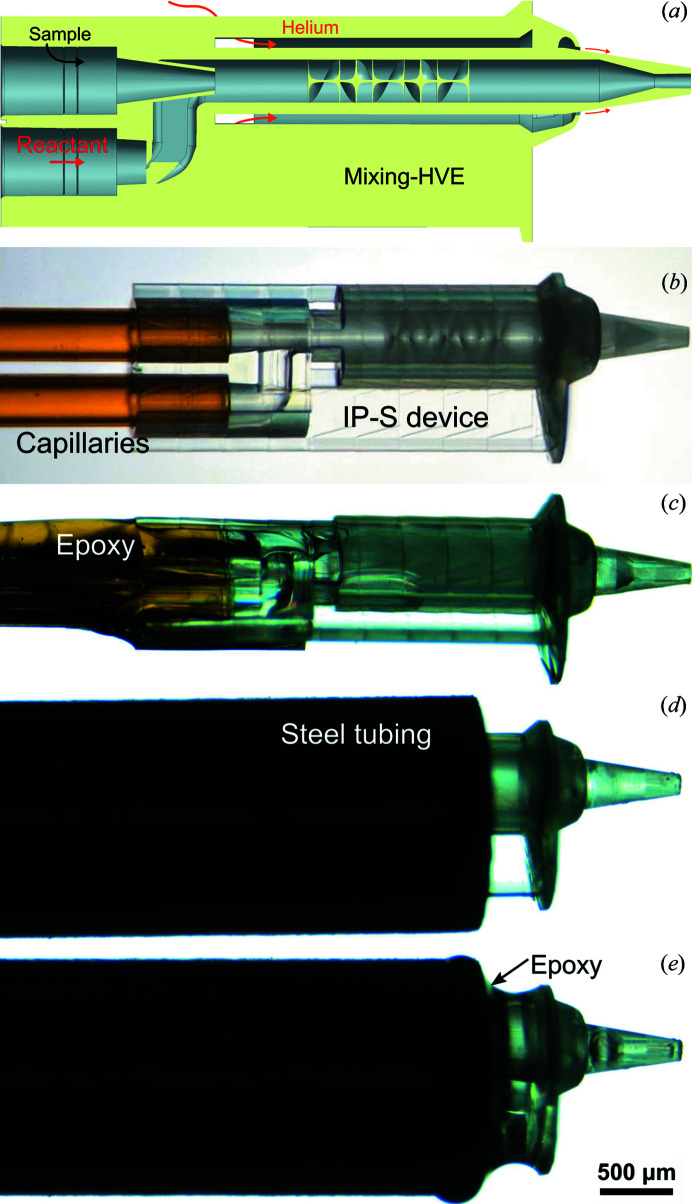
(*a*) Cross-sectional schematic of the 2PP-3D-printed mix-and-extrude device. (*b*)–(*e*) Microscopy images showing the assembly of the mixing-HVE tip with two fluid-feeding fused silica capillaries. (*b*) Two capillaries, each with 250 µm ID, are inserted into the 3D-printed mixing-HVE access ports and (*c*) glued with ep­oxy glue. (*d*) The capillaries (un-glued end) are fiddled through a 0.046′′ ID steel tubing (OD = 1/16′′), which is pulled over the mixing section of the device. (*e*) A small amount of slightly viscous ep­oxy glue (cured for 1 min) is added onto the gap with a clean capillary or metal wire to secure IP-S to the steel and provide gas tightness.

The mixer provides a dual-inlet section that accepts two capillaries and allows the convergence of two fluid channels via overlapping concentric cones [Fig. 1 ▸(*a*)]. At the start of the mixing channel, the main to side channel diameter ratio is 100:231.7 µm. Due to the centred sample inlet and the 3D hydro­dynamic flow-focusing geometry, a wall contact of the sample is initially (throughout the 500 µm in the downstream direction) prevented. The total length of the mixing channel is 2570 µm (design ‘J_7’). From this length, the initial 2070 µm have a 231.7 µm channel width, followed by a 300 µm-long tapering section [truncated cone which reduces the inner diameter (ID) down to 75 µm], leading to a 200 µm-long final section with a 75 µm inner diameter. For the geometry at the tip, the ID–OD–D (liquid channel diameter, gas orifice and distance between orifices) are chosen to be 75, 345 and 600 µm; therefore, 75 µm-wide streams are provided for the X-ray beam.

Inside the first section of the 231.7 µm-wide mixing channel, a modified mixing structure based on the ‘JKMH#10’ Kenics mixer from Knoška *et al.* (2020 ▸) is incorporated. With this, a series of six helical elements are introduced to the mixing channel for repeated flow splitting/stretching. The first blade is positioned 500 µm after the mixing initiation point (overlap of the liquid apertures).

With the 2570 µm-long mixing channel and a combined liquid flow rate of *Q*_total_ = 1.46 µl min^−1^ (*i.e.* 1.43 µl min^−1^ for the reactant and 0.03 µl min^−1^ for the sample), a retention time of 4.1 s before extrusion can be achieved within the 3D-printed part. This corresponds to a stream velocity of *v*_flow_ = 5.5 mm s^−1^ for the 75 µm-wide sample stream. Longer retention times require lower flow rates. For instance, as used during the diffraction data collection described below, a retention time of 18.2 s can be obtained with *Q*_total_ = 0.36 µl min^−1^ (exposed sample has a flow velocity *v*_flow_ = 1.3 mm s^−1^). Another design variation (‘J_8’) contains a shorter mixing channel (total length: 1685 µm). With the aforementioned flow rates, this shorter mixing-HVE allows retention times between 2.3 and 9.9 s. All 3D CAD files can be found in our online design repository: https://github.com/flmiot/EuXFEL-designs.

At the European XFEL, the X-ray pulses can arrive at 10 Hz, *i.e.* one pulse per train. From previous studies, it is known that flow velocities as low as *v* = 0.3 mm s^−1^ are sufficient for stable viscous sample extrusion while maintaining a pulse displacement of *ca* 30 µm on the sample and, as a result, effectively avoiding exposure of the same crystal with multiple X-ray pulses (Vakili *et al.*, 2022 ▸). Moreover, sample delivery to X-rays that arrive at higher pulse repetition rates, as seen at the FELs SACLA (60 Hz), PAL-XFEL (60 Hz), SwissFEL (100 Hz) and LCLS (120 Hz), can also be accommodated (Shimazu *et al.*, 2019 ▸; Lee *et al.*, 2020 ▸; Milne *et al.*, 2017 ▸; Wells *et al.*, 2022 ▸). However, for ≥100 Hz operation, a sample width reduction from 75 to 50 µm is highly recommended. At constant flow rates, this will increase the flow velocity by a factor of 2.2 which, in turn, facilitates viscous extrusion without the need for higher pump pressures.

### 2.2. Beam time injection setup

The SPB/SFX instrument of the European XFEL comprises two interaction regions: a high-vacuum upstream sample environment (interaction region upstream, IRU) and an in-helium downstream interaction region (interaction region downstream, IRD) with respect to the X-ray beam. The instrument setup at IRD provides high flexibility on sample deliveries, including the HVE injection setup. The injection rod funnel is attached to a helium atmosphere sample chamber, located between the vacuum out-coupling acoustic delay line (ADL) and detector (JUNGFRAU 4M), and the inserted nozzle rod is positioned using the *XYZ* motors of the goniometer support tower (Round *et al.*, in preparation).

**Figure 2:**
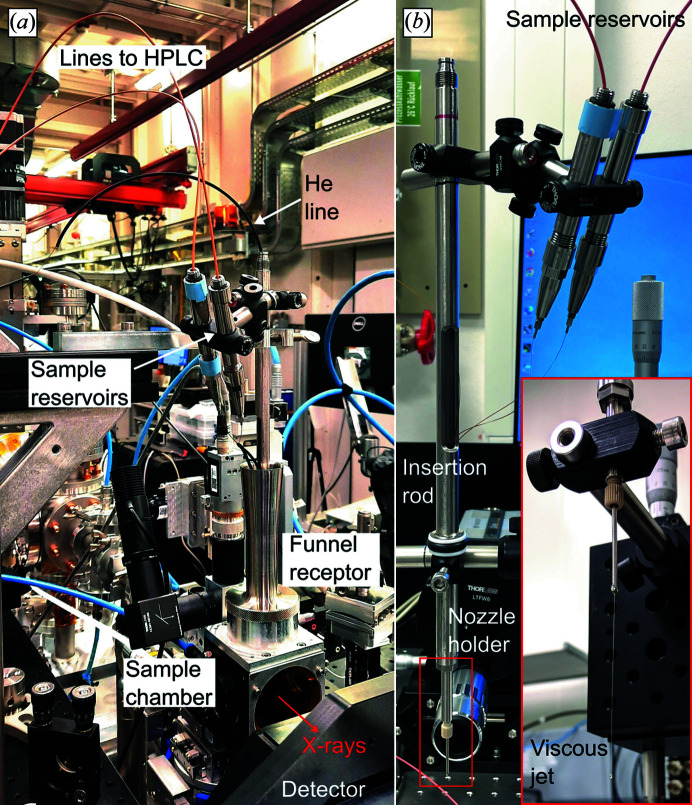
(*a*) Beamline injection setup surrounding the mixing-HVE device inside the helium-purged sample chamber at the SPB/SFX instrument and (*b*) in the laboratory test station during in-air operation.

The assembled nozzle, connected to capillaries and 1/16′′ OD steel tubing (IDEX, part No. U-145), was connected to the nozzle holder using an F333N fitting (IDEX) (Weierstall, 2014 ▸). A slit on the insertion rod [Fig. S1(*b*)] was created 10 cm above the o-ring to shorten the sample capillary length. The nozzle holder was screwed onto the metal rod and the rod was inserted into the IRD sample chamber, and then the capillaries were carefully taken through the slit and linked to the sample reservoirs of the HVEs using 0.015–0.0625′′ ID polyether ether ketone tubing and a custom No. 10–32 UNF steel fitting with a 1/16′′ through-hole (similar to part No. F-354, IDEX). ThorLab 0.5′′ mounting rods were used to mount the two HVE systems on the insertion rod to bridge the short distance to the nozzle [Fig. 2 ▸(*a*)]. The final capillary length was 30 cm, with an interior volume of 14.7 µl.

### 2.3. Sample preparation

**Figure 3:**
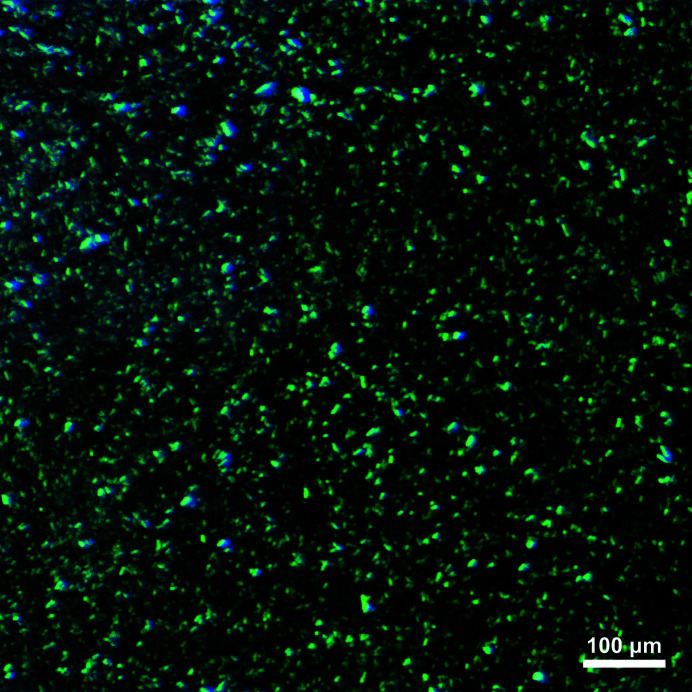
Stereomicroscopy image of iq-mEmerald crystals (*ca* 5 × 15 µm) embedded in LCP, used for the mix-and-extrude device characterization.

The preparation of the iq-mEmerald protein and crystal is described in the supporting information, and the following procedures were included to prepare samples suitable for HVE injection. LCP was prepared with a 7:3 ratio of monoolein to water using two gas-tight glass syringes and a coupler. To embed iq-mEmerald crystals (*ca* 5 × 15 µm) and CuCl_2_ in LCP, a 10%(*v*/*v*) crystal pellet and 20 m*M* CuCl_2_ solution were mixed with prepared LCP separately. To investigate the mixing inside the nozzle, iq-mEmerald protein embedded in LCP was used instead of protein crystals to provide a continuous fluorescence signal; a 65:35 ratio of monoolein and 10 mg ml^−1^ of iq-mEmerald protein in 50 m*M* Tris (pH 8.0) or 10 m*M* CuCl_2_ was mixed and resulted in 130 µ*M* protein and 3.5 m*M* CuCl_2_ in LCP, respectively (Fig. 3 ▸). The prepared samples were loaded into the HVE sample reservoirs and prepared for injection (Han *et al.*, 2021 ▸).

The samples were extruded using two HPLC pumps (Shimadzu, LC-20AD XR) connected to the two HVE injector systems (Weierstall *et al.*, 2014 ▸). The pumps were first run rapidly to fill the capillary with samples (up to 2.14 µl min^−1^). When the extruded sample volume was determined to be around 13 µl, shortly before the samples reached the nozzle, the flow rates were reduced to the desired values.

## 3. Results and discussion

### 3.1. Device characterization

We investigated the fluorescence quenching of iq-mEmerald protein in LCP mixed with copper ions for characterization of the mix-and-extrude nozzle on account of its facile visualization by means of optical microscopy. Moreover, the reaction time is so fast that it can be ignored in the mixing time scale of our nozzle, and the diffusion time of copper ions is expected to be fast compared with that of other ligands. The mixing ratio of crystal and copper ions was fixed to 1:3 so that the concentration of copper does not affect the quenching time and fluorescence intensity. The retention time for the characterization was triggered between 2.3 and 7.6 s (Fig. S4).

The same injection test was performed under a fluorescence microscope for quenching observation in iq-mEmerald crystals in LCP at high resolution (Movie S1 of the supporting information). Independently of the retention time, we could observe fluorescence quenching of mixed species within the jet, meaning that the diffusion of copper ions in LCP and the quenching reaction in iq-mEmerald crystals happened in less than 2.3 s. The diffusion and the reaction of the test system was very fast, and thus the fluorescence signal disappeared even before the sample was extruded from the nozzle. To measure how fast this diffusion and reaction happens within the mixing device, we reduced the intrinsic fluorescence of the IP-S photoresist by curing the device in a UV (λ = 385 nm) chamber (*XYZ* printing, 3UD10XEU01K) for 1 h followed by hard-baking at 80°C for 3 h.

**Figure 4:**
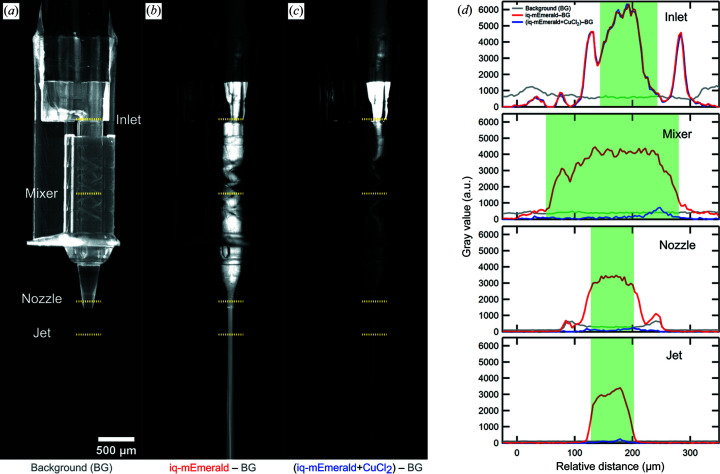
Observation of fluorescence quenching inside the 3D-printed mixing-HVE device. (*a*) Fluorescence microscopy image of the empty device which serves as the background image. (*b*) Background-corrected image of the sample, *i.e.* iq-mEmerald protein (130 µ*M*) in LCP flowing at 0.36 µl min^−1^ inside the device. (*c*) Background-corrected image showing the mixing of iq-mEmerald (130 µ*M*) and CuCl_2_ (3.5 m*M*) entering with a 1:3 flow rate ratio (0.36 and 1.07 µl min^−1^, respectively). The resulting flow velocity of the extruded mixture is 5.4 mm s^−1^ and the total retention time amounts to *ca* 4.4 s. (*d*) Pixel intensity profiles from the 16-bit fluorescence images (relative grey values extracted via *ImageJ* 1.53k) for four downstream positions: inside the 100 µm-wide sample orifice (inlet), inside the 231.7 µm-wide mixing channel within the Kenics section, 1 mm downstream from the inlet (mixer), within the final 75 µm-wide section of the device material before extrusion (nozzle), and in air, 300 µm away from the nozzle tip (jet). The line positions are denoted with the yellow dotted lines in (*a*). The green boxes in (*d*) indicate the geometric restrictions of the device/jet, which are the channel widths and jet diameter, respectively.

Fig. 4 ▸(*d*) depicts the spatial/temporal evolution of the fluorescence signal at four downstream positions: inlet, mixer, nozzle and jet. Each plot shows the extracted grey value intensity from the 2D fluorescence microscopy image as a function of the channel width [indicated with yellow dotted lines in Figs. 4 ▸(*a*)–4(*c*)]. At the ‘inlet’, where the two liquid channels overlap, the mixing time is equal to 0, and hence the mixture (blue) still has the same signal as pure protein (red). The fluorescence signal at the ‘mixer’ position corresponding to a mixing time of 1.8 s (based on the distance travelled of 1.02 mm) decreases to *ca* 10% of the initial 130 µ*M* concentration at the *t*_0_ position and further downstream; as more time passes, there is no significant fluorescence signal inside the nozzle tip or in the free-flowing jet, indicating full quenching by mixing.

### 3.2. Diffusion in LCP

One of the biggest challenges in time-resolved serial crystallography, which determines distinct intermediate structures throughout the reaction, remains the rapid mixing of microcrystals with ligands (Brändén & Neutze, 2021 ▸; Schmidt, 2013 ▸). Even in investigations using aqueous solutions, the diffusion time of the ligand to the crystal centre varies according to the crystal size and packing, *i.e.* crystals that are smaller in size or with a higher water content per unit cell are unquestionably better candidates for time-resolved crystallography, as the ligand diffusion time would be decreased for such crystals. For membrane protein crystals in LCP, diffusion is even more challenging. LCP has a similar packing structure to crystals, with the exception of a wider water channel than protein crystals. In addition, the inner diameter of our device mixing channel is 231.7 µm, which increases the diffusion time by 3.1 s for oxygen and 13.2 s for glucose by calculation if the ligands are assumed to diffuse directly into the centre of the LCP stream (Atkins & Paula, 2006 ▸). To reduce this diffusion time caused by the width of the stream, a Kenics architecture was employed in our mixing channel to support the mixing of crystal and ligand in LCP. With the series of helical elements for repeated flow splitting/stretching, we exponentially increase the diffusive interface with each additional element. Mixing time point uniformity/dispersion becomes more dependent on spatial location along the mixer and highly insensitive to flow rate fluctuations. This feature is in contrast to flow-focusing designs that tune diffusion distances through flow rate differentials. Even with these efforts to obtain discrete intermediates by reducing diffusion time, we are still likely to observe a mixture of multiple intermediates by mixing with this device. With current advancements in data analysis (*e.g.* *Xtrapol8*; De Zitter *et al.*, 2022 ▸), it is projected that mixed diffraction data of multiple states can be separated.

Owing to the complexity of the LCP structure and membrane protein, it is difficult to predict the diffusion and reaction time of ligand binding in LCP; for instance, if the ligand is diffused in an aqueous channel and the binding site of the membrane protein is located in an aqueous channel, the diffusion time would be comparable to that in aqueous solution. Alternatively, if binding occurs in the lipid, it is probably too time consuming to be studied using this type of device. Therefore, we assume that this mixing-HVE is suited for the examination of reactions taking place on the cell/membrane surface, which is what most current drugs targeting membrane proteins bind to (Yin & Flynn, 2016 ▸). However, due to the overall unpredictability of reaction in LCP, it is highly recommended to characterize the reactions in LCP prior to X-ray diffraction using different biochemical and biophysical techniques in order to confirm that the reaction takes place on a timescale compatible with the operation of these nozzles.

### 3.3. Time-resolved X-ray diffraction

To demonstrate the performance of the device under real life serial membrane protein crystallography conditions/requirements, we collected pre/post-mixing diffraction patterns of GPCR (G protein-coupled receptor) membrane protein crystals in LCP on the SPB/SFX instrument of the European XFEL (experiment No. 2826) using a photon energy of 12.4 keV, an X-ray pulse repetition rate of 10 Hz and a beam size of *ca* 2 × 2 µm. The pulse energy was 2.2 mJ and the pulse duration was *ca* 100 fs. The detector (JUNGFRAU 4M) was positioned 120 mm from the sample stream.

**Figure 5:**
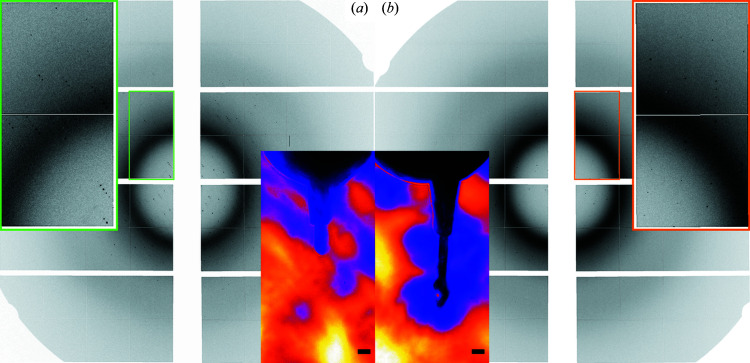
Diffraction patterns of membrane protein crystals (*a*) without mixing and (*b*) after mixing with ligand (*t* ≃ 18.5 s) collected with the JUNGFRAU 4M detector (pixel size is 75 × 75 µm) at room temperature (experiment No. 2826). The detector consists of eight front-end modules, each being 80 × 40 mm (1024 × 512 pixels) large. The prominent ring arises from monoolein scattering at 4.5 Å resolution. In the insets, the beamline side-view microscopy images show the respective viscous extrusion from the nozzle tip (the scale bar denotes 100 µm).

For injection, a co-flowing sheath of helium gas was used and the supply pressure was adjusted to 140 psi (corresponding to 24 mg min^−1^) to maintain a stable sample flow. The distance from the nozzle tip to the X-ray interaction was *ca* 200 µm. For *in situ* mixing, a combined flow rate of 0.36 µl min^−1^, *i.e.* 0.11 µl min^−1^ (sample in LCP) + 0.25 µl min^−1^ (ligand in LCP), with associated pressures of *ca* 450 and 420 psi at the HPLCs, was applied. This led to a flow velocity of 1.35 mm s^−1^ for the extruded mixture (sample width = 75 µm) and a probed time point of *ca* 18.5 s. The un-mixed sample was pumped with a flow rate of 0.36 µl min^−1^ (*i.e.* 1.35 mm s^−1^) with an associated pressure of *ca* 480 psi at the HPLC. Fig. 5 ▸ shows exemplary diffraction patterns exhibiting strong reflections from the crystals in both states. Details on the sample and data statistics are currently being prepared for a forthcoming article.

### 3.4. Outlook

The current setup, which utilizes externally positioned fluid-feeding capillaries, can be susceptible to capillary rupture. Moreover, sample-conserving inner diameters below the 250 µm used here can be prone to generating high pressures during injection. Note that the capillary length of 30 cm used generated a void volume of 14.7 µl. Especially when considering the difficulties of membrane protein sample preparation and the available sample reservoir capacities of 40 and 120 µl, this sample loss might not be negligible. To reduce the length of the capillaries, a more compact mixing-HVE composed of two hydraulic systems within a single, protective injector rod is desired. In addition, the installation of injection infrastructure with a large footprint should be avoided to prevent interference with other significant instrumentation (detector, laser setup, electronics, optics *etc*.) already present at the beamline.

The design of another type of HVE described in the recent publication by Shimazu *et al.* (2019 ▸) allows a capillary connection to the sample reservoir without the need for direct screwing. Thus, we assume that an adaptation of their design would enable the advancement of a compact mixing-HVE utilizing very short capillaries.

As described above, the diffusion time of diluted species in LCP media is difficult to predict. To cover a broader range of diffusion and reaction time, modification of the device design is under consideration. For shorter retention time, the length of the mixer can be reduced. To access longer retention times, a modular assembly approach can be pursued in which the 3D-printed mixer and nozzle are connected by a capillary extension of customized length. In addition, once the compact mixing-HVE is commissioned and shorter capillaries can be used, the ID of capillaries and mixer/nozzle can be reduced because pressure build-up due to long capillaries is no longer a concern. Then, even higher sample flow rates can be applied to the system: with faster sample movement in the mixer, the retention time can be reduced further.

## 4. Conclusions

We have fabricated and characterized a mix-and-extrude nozzle for time-resolved protein serial crystallography. Fluorescence quenching by mixing iq-mEmerald crystals with copper ions was observed on a short timescale, indicating that the diffusion in LCP occurred in a negligible time compared with the retention time of the mixer. With current designs of the device, a retention time between *ca* 2 and 20 s can be triggered, and injectors aiming for shorter and longer retention times can be prepared with minor design modification. The first EuXFEL user experiment using this nozzle in a helium environment at atmospheric pressure has been conducted and the manuscript describing the result is currently in preparation. We anticipate that this mix-and-extrude nozzle will be widely used for mixing experiments of viscous media containing, for instance, membrane protein crystals at synchrotrons and FELs, hence, efforts to create a mixing-HVE as a single compact system are ongoing.

## 5. Related literature

The following references, not cited in the main body of the paper, are cited in the supporting information: Samarkina *et al.* (2009 ▸); Yu *et al.* (2014 ▸).

## Supplementary Material

Movie S1: injection test under a fluorescence microscope for quenching in iq-mEmerald crystals in LCP at high resolution. DOI: 10.1107/S1600576723004405/jo5080sup1.mp4

Methods and supporting figures. DOI: 10.1107/S1600576723004405/jo5080sup2.pdf
