# Supplementary material for: Mix-and-extrude: high-viscosity sample injection towards time-resolved protein crystallography
Source: J Appl Crystallogr. 2023 Jun 12;56(Pt 4):1038–45. doi: 10.1107/S1600576723004405 (PMC10405586; doi:10.1107/S1600576723004405)
Supplement: Supplementary file 2 [file j-56-01038-sup2.pdf]

JOURNAL OF
APPLIED
CRYSTALLOGRAPHY

Volume 56 (2023)

Supporting information for article:

Mix-and-extrude: high-viscosity sample injection towards time-resolved protein crystallography

Mohammad Vakili, Huijong Han, Christina Schmidt, Agnieszka Wrona, Marco Kloos, Iñaki de Diego, Katerina Dörner, Tian Geng, Chan Kim, Faisal Koua, Diogo Melo, Mathieu Rappas, Adam Round, Ekaterina Round, Marcin Sikorski, Joana Valerio, Tiankun Zhou, Kristina Lorenzen and Joachim Schulz

S1. Materials and Methods

S1.1. Device fabrication and assembly

The 2PP-based microfabrication followed general guidelines detailed elsewhere (Knoška *et al.*, 2020).

3D geometries were designed in AutoCAD (Autodesk) or NX (Siemens) and exported as STL formats. Using DeScribe (Nanoscribe), the STL-based 3D designs were converted to print-job instructions (GWL). For fast printing times, slicing distances of 2 µm, hatching distances of 0.7 µm, and block sizes of 285/285/299 µm (x/y/z) with 15° block shear angles, 2 µm block overlaps and 1 µm layer overlaps were chosen.

The devices (solid volumes) were then printed with the IP-S photoresist using the Nanoscribe Photonic Professional GT equipped with a 25× objective lens (Zeiss) in upwards direction (+z) with alternating hatch lines and in the dip-in mode. IP-S was deposited onto an indium tin oxide (ITO) coated glass slide. The laser power was 100% (*i.e.* 156 mW exiting the laser source, 70 mW arriving at the objective), and print speeds were 100,000 µm s^{-1}. With these parameters, the printing time for one mixing-HVE device was 3 h for J_7 and 2 h for J_8, respectively. It is worth noting that the shorter design allows the printing of multiple devices in one batch (*i.e.* one glass slide) which reduces the overall device fabrication time.

For the device assembly, two fused silica capillaries (Polymicro, OD 360 µm, ID 250 µm), each 30 cm in length, were inserted into the liquid access ports of the 3D printed IP-S device and glued with freshly mixed (low-viscous) epoxy glue (Devcon 5-minute epoxy for general purpose) (Fig. 1). It was crucial that the amount of applied glue does not exceed the total width of the 2PP–3D printed part. Otherwise, the steel tubing cannot be imposed on the IP-S–capillary assembly. To minimize sample waste due to lengthy capillaries, one might consider the connecting of a cartridge with pure LCP after the sample is run empty to push out the remaining crystal from the capillary.

After epoxy curing for 12 h at RT, the two capillaries were run through a steel tubing with 0.046 in ID (IDEX, part no. U-145), which was brought into close proximity to the ‘collar’ of the IP-S tip at the other end. A small gap of the width of a capillary was left between steel tubing and the nozzle’s ‘collar’ to allow the deposition of a small amount of slightly viscous epoxy glue (cured for 1 min). After thus bridging the steel and IP-S with strong epoxy bonds, the steel tubing was connected to a T-union (P-728 or custom variant) using a #10-32 UNF (IDEX, part no. F-333N) fitting. The through-hole of the T-union must be larger than 1 mm to allow room for two capillaries. The opposite port of the T-union (port #2, *i.e.* where the two capillaries exit) allows connection at the Interaction Region Downstream (IRD) at the SPB/SFX instrument via insertion rod (Fig. S1A&B). (Round *et al.*, in preparation) Alternatively, mounting can occur using the nozzle holder (Weierstall, 2014) and standard mounting parts (Fig. S1C). The exiting fluid-feeding capillaries can then be connected to

either pressure-driven syringe pumps or the ASU injector system for pumping high-viscous samples (Weierstall *et al.*, 2014).

The third port of the T-union is used to connect another capillary for introducing pressurized helium using a flexible tubing sleeve (F-242, 0.0155 in ID) in combination with a F-354 nut and a LT-135 ferrule (all from IDEX). In this manner, the helium enters the steel tubing and runs through the 3D printed pockets of the injector to sheath the mixed liquid streams after extrusion (Fig. 2A). To make sure that the helium is not released through the second port, a dual-lumen sleeve (FEP MultiLumen, Zeus Industrial Products, Inc.) can be used as shown in Fig S1A. Alternatively, the third capillary for helium supply can be simply inserted directly into the back of the steel tubing (where the two liquid-feeding capillaries exit) and sealed with epoxy glue. Details on utilized injection hardware can be found elsewhere (Vakili *et al.*, 2022).

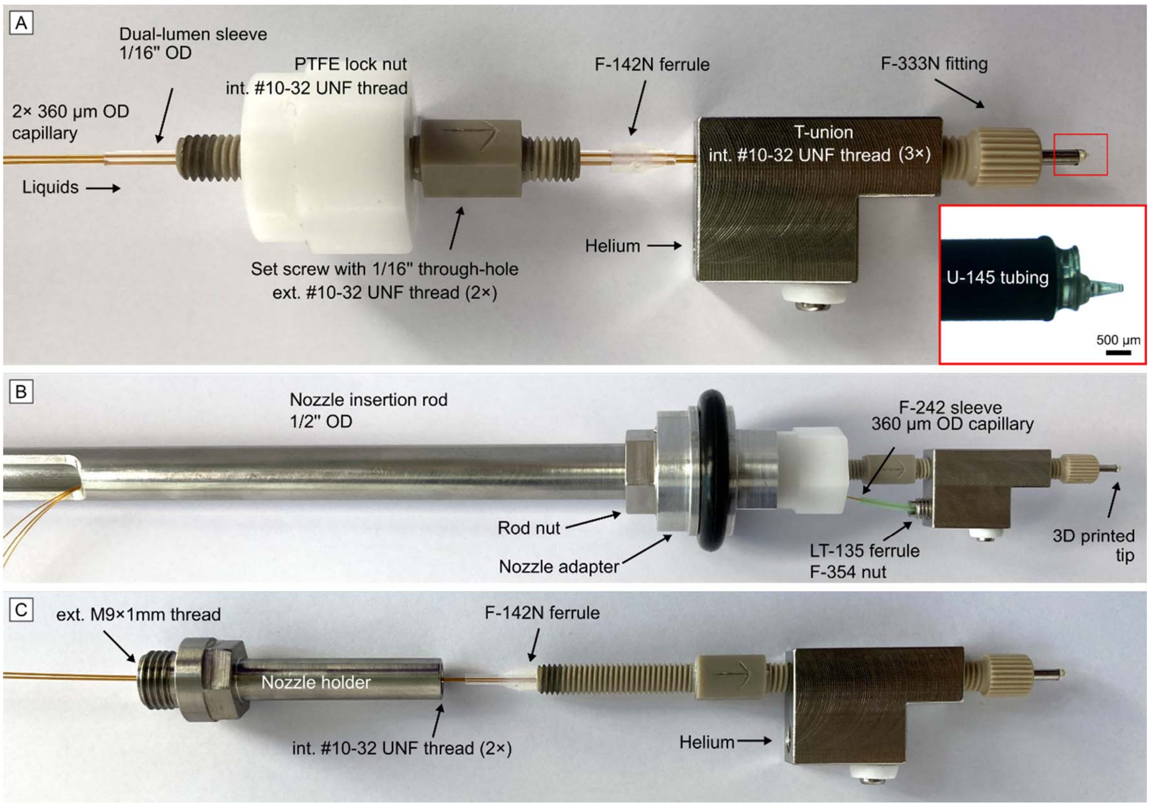

Figure S1 Photographs of the injector assembly surrounding the 3D printed mixing-HVE device. The use of wider ID capillaries (250 µm), allows the injector tip to be in ca. 30 cm distance from the feeding sample reservoirs (not depicted here). (A) Close-up of the injector system connected to a custom steel T-union with description of utilised fluid connection parts. For a uniform helium stream, which enters the third port of the T-union and exits through the injector tip, a dual-lumen sleeve, which encases two liquid capillaries at once, is used. (B) Depiction of the injector system after connection to a 30 cm long insertion rod for in-vacuum/in-helium beamline installations at the IRD@SPB/SFX. The connective nozzle adapter contains an internal #10-32 UNF thread for device mounting and an external groove for a 25×5.5mm O-ring for sealing the sample chamber. (C) Instead

of using the nozzle adapter, a combination of a dual-lumen sleeve, dual-fittings (#10-32 UNF threads) and the ‘nozzle holder’ can be used for in-air operation. Here, at the right end of the nozzle holder, the tan dual-fitting is inserted and the assembly continues as shown in (B).

S1.2. Preparation of iq-mEmerald microcrystals

Iq-mEmerald is a GFP-derivative designed and studied by Yu *et al.* (Yu *et al.*, 2014). It has an UV-Vis absorption maximum at 488 nm and an emission maximum at 512 nm. The pET17b-iq-mEmerald expression vector was purchased from Biocat GmbH (Heidelberg, Germany). One colony of *Escherichia coli* BL21 DE3 pET17b-iq-mEmerald was transferred into 50 mL LB containing 100 µg mL^{-1} Ampicillin and was incubated at 37 °C for 16 h at 180 rpm. The next day, the OD_{600nm} was adjusted to 0.1 for 1 L LB containing 100 µg mL^{-1} Ampicillin and incubated at 37 °C and 180 rpm until the OD_{600nm} reached 0.6. Recombinant expression of iq-mEmerald was induced by adding 0.5 mM IPTG. Cultures were cultivated for 16 h at 18 °C and 180 rpm. Cells were harvested at 8000× g for 1 h at 4 °C and cell pellets were stored at -80 °C. The purification was adapted from Samarkina *et al.* (Samarkina *et al.*, 2009). Cell lysis was performed after resuspending the pellet in lysis buffer (20 mM Tris, pH 7.8, 150 mM NaCl) using a sonicator. After centrifugation for 15 min at RT and 11000 rpm, the supernatant was heated up for 15 min at 65 °C. Reaction tubes were spun down for 15 min at 11,000 rpm and the supernatant was collected. 10 mL of cell lysate was rapidly mixed with 3 mL of 5M NaCl and 23.3 mL saturated $(NH_4)_2SO_4$ (pH 7.8). 12 mL of 96% Ethanol was added instantly and vortexed for 30 sec. Samples were spun down for 7 min at RT with 3000× g. Iq-mEmerald is present in the organic phase which is carefully removed and diluted to 20% $(NH_4)_2SO_4$ saturated solution in 20 mM Tris, pH 7.8. The sample was filtered with a 0.2 µm filter and subjected to a HIC (hydrophobic interaction chromatography) column that was equilibrated with 20 mM Tris (pH 7.8), 20% $(NH_4)_2SO_4$ saturation. Sample was eluted over 20 column volumes of elution buffer (20 mM Tris, pH 7.8). The purified protein was concentrated to 50 mg mL^{-1}.

For batch crystallization, 500 µL protein solution (50 mg mL^{-1}) were mixed with 500 µL of 3 M $(NH_4)_2SO_4$, 50 mM Tris (pH 8.0) as well as 5 µL seed-stock and vortexed for 30 sec. After 1 min, additional 500 µL of 2.5 M $(NH_4)_2SO_4$, 50 mM Tris (pH 8.0) were added and vortexed briefly. Crystals grew overnight to 5×15 µm. Crystals were filtered with a 20 µm gravity filter before being embedded in LCP.

S1.3. Injection test station with fluorescence microscopy setup

Fluorescence microscopy was conducted with a Zyla 4.2 sCMOS camera equipped with a 2× objective (Mitutoyo, #46-142) leading to a pixel size of 3.6 µm. As a light source, a fiber-coupled LED (ThorLabs, M490F3), λ = 490 nm (FWHM = 26 nm), together with a fluorescence imaging filter set, composed of a 475/35 nm FITC excitation filter (MF475-35) and a 525/39 nm GFP emission filter (MF525-39), has been used (Fig. S3).

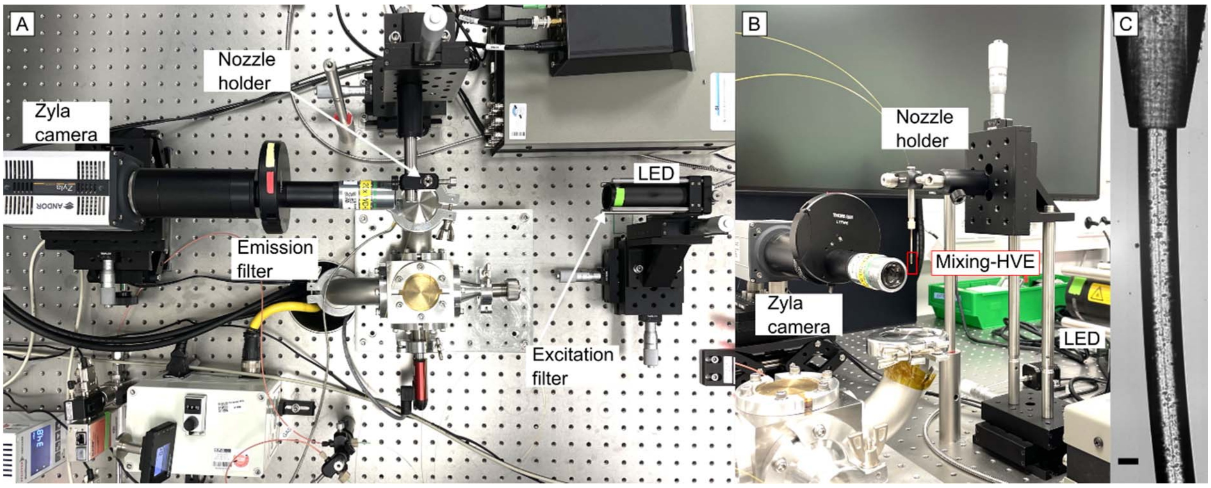

Figure S2 Injection test station setup from (A) the top view and (B) from the side view depicting the LED light source with excitation filter and Zyla camera with emission filter. (C) Detailed view of the mixing-HVE tip (here with a 100 µm ID). The exemplary microscopy image (125×615 pixels, pixel size ca. 1.9 µm) depicts the dual-extrusion of LCP media, each delivering *ca.* 5 µm wide lysozyme crystals with a total flow velocity of 1.3 mm s^{-1} (as used for detector calibration). The scale bar denotes 50 µm.

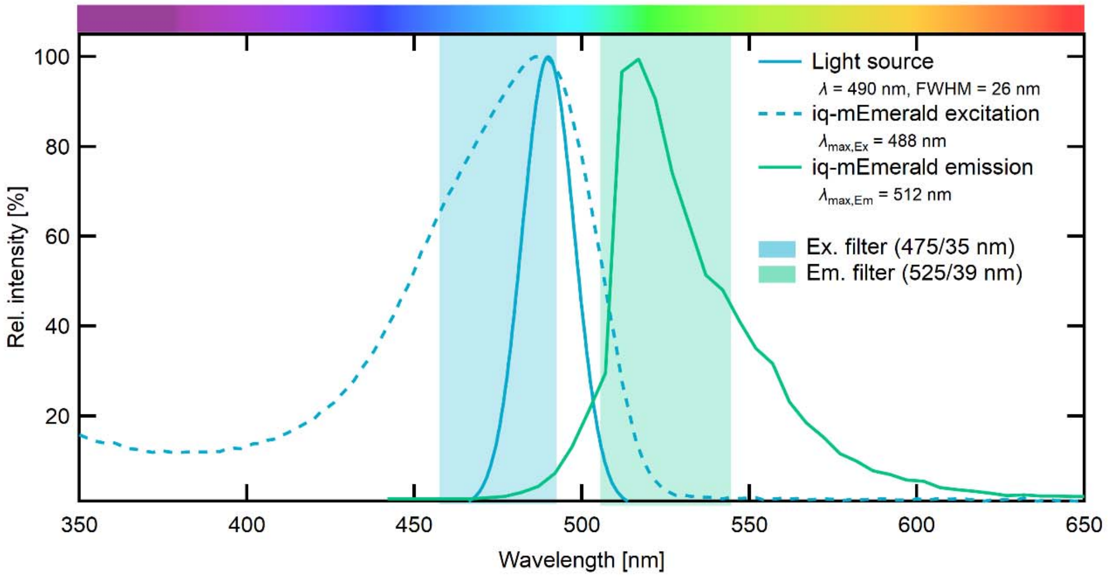

Figure S3 UV-Vis absorbance and emission spectra for the iq-mEmerald crystals with indicated excitation/emission filter range of the imaging system (see S1.3.) and spectral line of the 490 nm (FWHM = 26 nm) LED light source. The absorption spectrum of the protein in solution was recorded with a Tecan Spark 10M microplate reader. The emission spectrum was acquired from a single crystal in 5 nm steps on a Nikon AX-R confocal microscope equipping a DUX-VB4 detector, using the 405, 445, and 488 nm channels (LUA-S6 laser unit) for excitation and the spectral acquisition mode from the Nikon NIS-Elements AR 5.41.01 software.

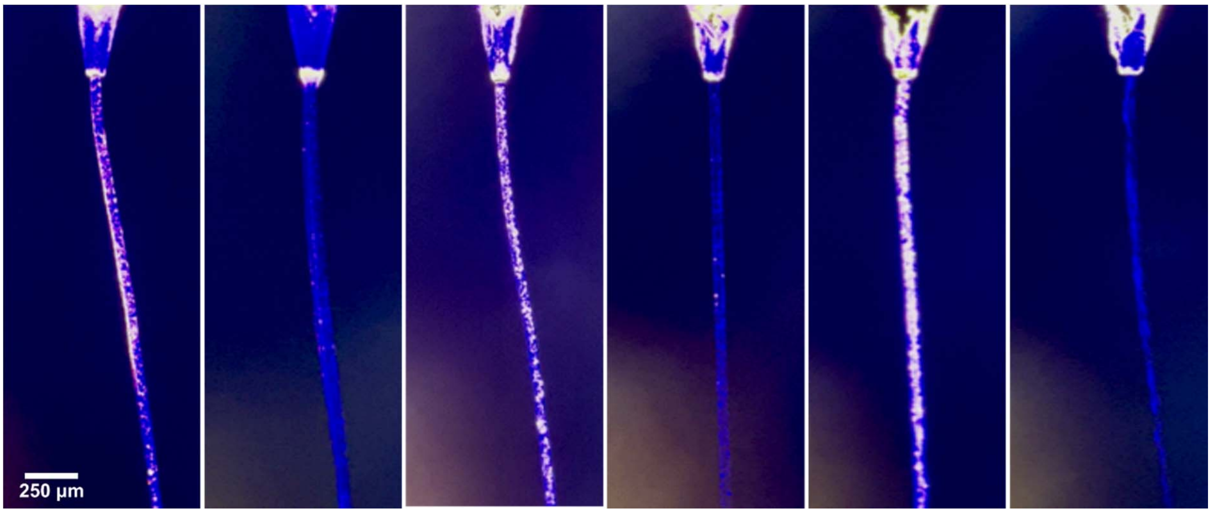

Figure S4 Fluorescence microscopy images taken at the setup described in S1.3. depicting (left) the extrusion of iq-mEmerald crystals (*ca.* 5×15 µm) and (right) fluorescence quenching of iq-mEmerald crystals by mixing with Cu^{2+} with variation of the flow velocity (thus retention time in the mixer) (22.5, 4.5, and 2.2 seconds, respectively).

S1.4. Fluorescence videography setup

12-bit frames (2048×1048 pixels, 2fps) were recorded on a Nikon AX-R confocal microscope equipped with a Plan Apo lambda 20× objective (NA = 0.75), using the resonant bidirectional scanner mode and a pinhole size set to 34.9 µm. A 488 nm laser was used for excitation with an emission gate between 499 nm and 551 nm. Image sequence was processed to reduce the shot noise from the resonant scanner using the deep learning-based Denoise.ai algorithm from the Nikon NIS-Elements AR 5.41.01 software, background-corrected and exported to an 8-Bit RGB compressed MP4 video file.

Movie S1.mp4

Movie S1 Fluorescence microscopy video (see S1.4.) showing fluorescent iq-mEmerald crystals in LCP while being extruded from the tip of the nozzle and subsequently quenched by flow start of $CuCl_2$.
